# Preemptive analgesic effectiveness of single dose intravenous ibuprofen in infants undergoing cleft palate repair: a randomized controlled trial

**DOI:** 10.1186/s12887-021-02907-6

**Published:** 2021-10-22

**Authors:** Zhe Zhe Peng, Yan Ting Wang, Ma Zhong Zhang, Ji Jian Zheng, Jie Hu, Wan Ru Zhou, Ying Sun

**Affiliations:** 1 Department of Anesthesiology, Shanghai Children’s Medical Center, School of Medicine, Shanghai Jiao Tong University, Shanghai, China; 2 School of Clinical Medicine, Xuzhou Medical University, Xuzhou, China

**Keywords:** Ibuprofen, Cleft palate, Infant, Pain

## Abstract

**Background:**

Correction surgery for cleft palate is recommended between 9 and 18 months of age. Patients suffer from acute pain after palatoplasty. Clinicians are hesitant to use opioids for analgesia concerning the potential high risk of respiratory adverse events. Intravenous ibuprofen perhaps be a suitable adjuvant to pain relief. We try to assess whether preoperative administration of intravenous ibuprofen can decrease opioid requirements following cleft palate repair in infants.

**Methods:**

This single center prospective randomized clinical trial was performed from February to April 2021 at Department of Anesthesiology in Shanghai Children’s Medical Center. Forty patients ASA I-II, aged 9–24 months with isolated cleft palate and undergoing palatoplasty were randomized in a 1:1 ratio to receive either a single dose of 10 mg/kg ibuprofen intravenously or normal saline at induction. Children and infants postoperative pain scale (CHIPPS) was used for pain assessment. Those patients CHIPPS pain score equal or higher than 4 received analgesic rescue with titrating intravenous fentanyl 0.5 μg/kg and repeated in 10 min if required. The primary outcome was the amount of postoperative fentanyl used for rescue analgesia in postanesthesia care unit (PACU).

**Results:**

Patients (*n* = 20 in each group) in IV-Ibuprofen group required less postoperative fentanyl than those in placebo group (*p*<0.001). There was no significant difference between two groups in first rescue analgesia time (*p* = 0.079) and surgical blood loss (*p* = 0.194). No incidence of obvious adverse events had been found within the first 24 h after surgery in both groups.

**Conclusions:**

Preemptive intravenous administration ibuprofen 10 mg/kg at induction had a significant opioid sparing effect in early postoperative period without obvious adverse effects in infants undergoing palatoplasty.

**Trial registration:**

CHICTR, CTR2100043718, 27/02/2021 http://www.chictr.org.cn/showproj.aspx?proj=122187

## Background

Cleft palate (CL) is one of the most common congenital craniofacial birth defects in human. The prevalence of abnormality is from 3.4 to 22.4 per 10,000 births and has geographic variation, the highest prevalence is among Asians and Latin Americans [1]. Palatoplasty is recommended between 9 and 18 months of age [2], in order to minimize the further speech abnormalities and optimize the facial growth. Surgical correction for velopharyngeal usually induces to significantly acute postoperative pain. In approximately three decades ago, acute pain during this period was ignored due to the bias regarding the immaturity of their nervous system and inability to verbally express their feeling of pain. However, the misconception is gradually corrected as the enhancement for understanding of neurobiology pathway for this group.

After cleft palate surgery, the pharyngeal space is reduced dramatically. The child is at risk for acute upper airway obstruction due to upper airway narrowing, bleeding and edema [3–5]. Besides, the residual anesthetic effects may worsen the condition. Naturally, the main analgesia goal for cleft palate repair is to provide an effective pain relief with minimal risk of airway management. Available modality of postoperative analgesia usually entails a combination of opioids and/or non-opioid analgesics. Opioids are traditionally regarded as the best choice for their positive therapeutic effect on postoperative pain. However, the use of opioids in infants raises justifiable concerns regarding postoperative respiratory depression especially in this special population. Nonsteroidal anti-inflammatory drugs (NSAIDs) are alternative non-opioid analgesic choices. In clinic, NSAIDs are used for preemptive analgesia which focuses on pain management by preventing central sensitization according to their potential in inhibiting cycloxygenase enzyme to limit the inflammatory cascade [6, 7].

Using nonsteroidal postoperatively for palate surgery is a standard choice according to a survey in the UK and Republic of Ireland [8], but the therapeutic effect is contradictive. Bremerich et al. compared three doses of rectal acetaminophen (10, 20, and 40 mg/kg) in children undergoing cleft palate repair and concluded that the practice was lack of proof in opioid sparing efficacy [9]. This might be attributed to the slow and variable absorption of acetaminophen by the rectal route [10]. Dosage form also seemed to be the keypoint, as another research suggested that intravenous acetaminophen at 12.5 mg/kg was associated with opioid-sparing effects [11]. The only two injectable NSAIDs approved for intravenous use by Food and Drug Administration (FDA) are ibuprofen injection and ketorolac [12]. Unlike intravenous ketorolac, which is recommended only for patients aged above 2 years [13], the ibuprofen injection is widely used nearly across all age groups. Still, some clinicians are hesitant to use NSAIDs in procedure concerning the potential for hemorrhage. Previous study assessed the safety and efficacy effect of ibuprofen injection on pain relief after tonsillectomy surgery and concluded a significant opioid sparing effect without significantly increasing incidence of postoperative adverse events for children [14]. As for infants, there is a lack of clinical data abundance for the effect of the injection on postoperative pain at present.

We hypothesize that preoperative administration of intravenous ibuprofen can decrease opioid requirements following cleft palate repair in infants.

## Methods

### Study design and setting

This single center prospective randomized clinical trial was approved by the Institutional Review Board of Shanghai Children’s Medical Center and registered in ClinicalTrials.gov (ChiCTR2100043718, 27/02/2021). It was performed from February to April 2021 in Shanghai Children’s Medical Center, the trial carried out in accordance with the declaration of Helsinki and good clinical practical guidelines, and the authors guaranteed the accuracy and completeness of the data and analysis of this paper.

### Participant

Patients suffered from isolated CL, American Society of anesthesiologists (ASA) physical status I or II, aged 9–24 months, and undergoing palatoplasty were recruited in the study. Patients were excluded for non-intravenous access, contraindications to any study drugs, significant cognitive disorder, active asthma, coagulation disorders, liver or renal dysfunction. Patients were also excluded from enrollment if they had taken analgesic less than 4 h prior to study drug administration.

### Randomization

Written consent was obtained from the parents, the eligible participants were assigned a written number on paper and tossed into box, the desired number was taken out of the box by drawing lots based on the assigned rank. Statistics specialist generated the random allocation sequence, a specified researcher enrolled participants, and assigned infants to two groups: IV-Ibuprofen group or Placebo group.

### Sample size

A sample size of 36 patients needed in order to provide at least 80% power to detect a 0.75 μg/kg dose reduction in fentanyl requirement between groups [14]. This research enrolled 40 participants to randomize in a 1:1 ratio into two groups (*n* = 20 in each group) considering data loss in practice.

### Standardized anesthesia procedure

Patients were induced with 8% sevoflurane in oxygen and air via face mask, followed by intravenous catheterization. Atropine 0.01 mg/kg and hemocoagulase 0.5 IU were administered intravenously before intubation. Tracheal intubation was facilitated intravenously with propofol 2 mg/kg, fentanyl 1 μg/kg, and rocuronium 0.6 mg/kg. Study medication was applied after intubation. Anesthesia was maintained with 1–2 minimum alveolar concentration sevoflurane in oxygen and air. All procedures were performed by the same surgeon. Prior to incision, local infiltration of surgical field with 0.5% lidocaine and epinephrine (1: 200000 or 5 μg/ml), up to 5 mg/kg (1 ml/kg) were performed. All patients received Sodium Lactate Ringer’s transfusion with 10 ml/kg/h intraoperatively. They were extubated when fully awake and sugammadex 2 mg/kg was injected routinely before extubation. Patients were transferred to PACU after tracheal extubation and discharged from PACU according to Aldrete score [15], which was a most widely used measurement tool for postanesthesia recovery that included gauging a patient’s consciousness, activity, respiration, and blood pressure. Aldrete score ≥ 9 was considered satisfactory.

### Administration of study medications

The administration of study medications was double blinded. Patients, investigators, and care providers were blinded to random allocation. Only the pharmacy staff was unblinded. Eligible patients were randomized in a 1:1 ratio to receive a single dose of either 10 mg/kg ibuprofen injection (IV-Ibuprofen group) or normal saline (Placebo group) immediately after intubation. The placebo was identical with ibuprofen injection in terms of volume, color, appearance, and packaging. Infusion was applied by a pump device set as 10 min and finished before incision. The choice of ibuprofen injected dose was based on previous research that showed peak level time obtained in cerebrospinal fluid was 30-40 min after injection of this dose [14, 16].

### Pain assessment and opioid administration

Acute postoperative pain in PACU was assessed by a same examiner every 10 min by using CHIPPS system [17], a five observational items scale included gauging a patient’s crying, facial expression, posture of the trunk, posture of the legs, motor restlessness, which was suitable for children aged 0 to 5 years and ranged from 0 to 10 (no pain to maximal). A pain score<4 of 10 was considered satisfactory. Pain scores of 4 and higher received analgesic rescue with intravenous fentanyl 0.5 μg/kg and repeated in 10 min if required. Every single dose fentanyl required to achieve satisfactory analgesia effect was titrated injection. The accumulative maximum dose for fentanyl was 2 μg/kg (4 boluses). Respiration rate, oxygen saturation, and heart rate were continuously monitored. The observational period in PACU was 60 min at least.

### Outcome variables

The primary outcome of the research was the amount of postoperative fentanyl used for rescue analgesia in PACU. Additional outcomes included intraoperative blood loss, patient pain scores rating, time to first dose of analgesic rescue medication, incidence of postoperative nausea, vomiting and any adverse events. The safety assessment was evaluated through comparison of treatment-emergent adverse events. Continuous monitoring of adverse events lasted for 24 h after surgery.

### Statistical analysis

All statistics were performed using SAS 9.1 (SAS Institute Inc., Cary, NC, USA) with a significance level at 0.05. The normal distribution of data was analyzed using Shapiro-Wilk test. All data were expressed as mean and standard deviations (SD), median and interquartile (IQR) or number of participants (%). Two-way repeated measures of analysis of variance were used to multiple comparison. Greenhouse-Geisser Epsilon was used for correction. Two tailed student’s t-test was used to compare all means for normally distributed data. Wilcoxon rank-sum test was used to perform nonparametric statistics. Categorical data were compared using Cochran-Artimage trend test and chi-square.

## Results

**Fig. 1:**
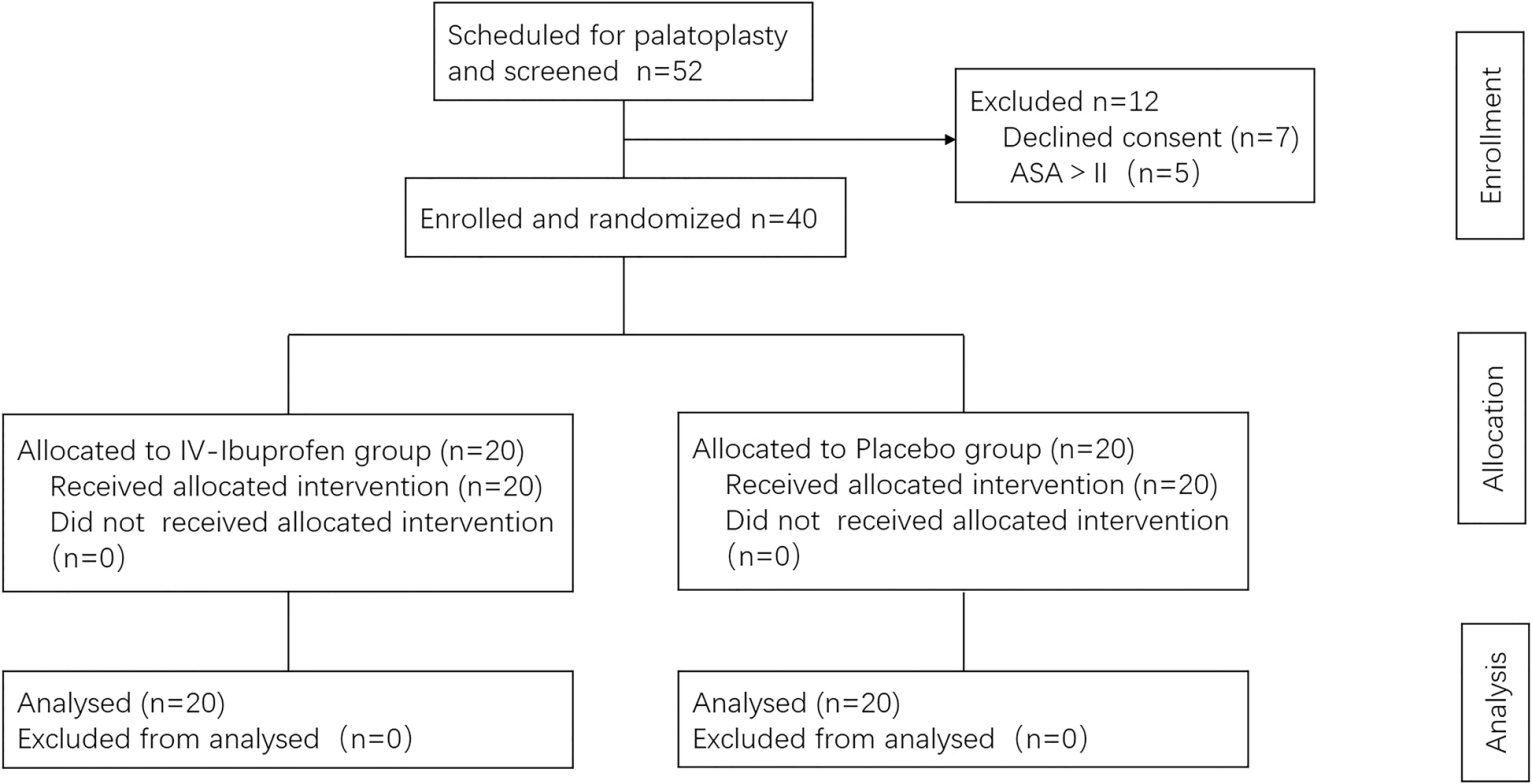
Consolidated Standards of Reporting Trials (CONSORT) diagram. Patients were enrolled and randomized in a 1:1 ratio into IV-Ibuprofen group and Placebo group; ASA, American Society of Anesthesiologists physical status classification

In this study, fifty-two patients with CL were scheduled and screened and forty patients were finally enrolled in the research (Fig. 1, CONSORT diagram). No data were loss. Veau class was used to evaluate the cleft palate according to morphologic basis [18], it classified palate clefts as four morphological forms. There were no significant intergroup differences in distribution of demographic factors and surgical characteristics (Table 1).

**Table 1:** Demographic Data and Surgical Characteristics

	IV-Ibuprofen group, *n* = 20	Placebo group, *n* = 20	*P* -value
Age (month)	13.8 ± 2.4	13.6 ± 1.7	0.674
Weight (kg)	10.1 ± 0.8	9.9 ± 1.1	0.442
Height (cm)	77.1 ± 3.8	76.6 ± 3.4	0.699
Sex (n)			0.744
Male	8	7	
Female	12	13	
Veau class (n)			0.478
I	3	6	
II	15	13	
III	2	1	
IV	0	0	
Surgery time (min)	54.3 ± 7.4	50.7 ± 6.1	0.097

Data were presented as n, mean ± SD; Veau class was a methodology to classified palate clefts as four morphological forms

The postoperative analgesic requirements for patients were summarized in Table 2. In the efficacy analysis, the number of patients received rescue analgesia therapy from IV-Ibuprofen group was significantly less than Placebo group (8 vs. 19, *p*<0.001). For those received postoperative analgesia treatment, patients from IV-Ibuprofen group also required less doses fentanyl 0,(0, 0.50) ug/kg than control patients (0.95 ± 0.42 μg/kg; *p*<0.001) significantly. Furthermore, four patients from IV-Ibuprofen group and six patients from Placebo group received one dose rescue administration treatment, respectively. Four patients from IV-Ibuprofen group and eight patients from Placebo group respectively received two doses rescue fentanyl therapy. There were 5 patients from Placebo group received 3 doses of postoperative rescue fentanyl while nobody from IV-Ibuprofen group received this rescue administration doses. None received 4 doses of rescue fentanyl treatment in both groups. Interestingly, the first rescue analgesia time for patients was statistically insignificant between two groups (*p* = 0.079).

**Table 2:** Postoperative analgesic requirements

	IV-Ibuprofen group, *n* = 20	Placebo group, *n* = 20	*P* -value
Number (%) of patients who received rescue fentanyl in PACU	8 (40.0%)	19 (95.0%)	<0.001
Rescue doses of fentanyl in PACU (ug/kg)			
mean ± SD	0.32 ± 0.44	0.95 ± 0.42	–
median (Q1, Q3)	0 (0,0.50)	1.00 (0.60,1.20)	<0.001
Frequency for patients who received fentanyl in PACU			<0.001
0 dose of fentanyl	12	1	
1 dose of fentanyl	4	6	
2 doses of fentanyl	4	8	
3 doses of fentanyl	0	5	
4 doses of fentanyl	0	0	
Total fentanyl dose in PACU (ug)			
Mean ± SD	3.20 ± 4.49	9.40 ± 4.49	–
Median (Q1, Q3)	0 (0,5.00)	10.00 (5.50,13.50)	<0.001
Time to first rescue analgesia (min)	16.75 ± 7.25	11.37 ± 6.16	0.079

Data were presented as n, percentage, mean ± SD or median (IQR)

**Fig. 2:**
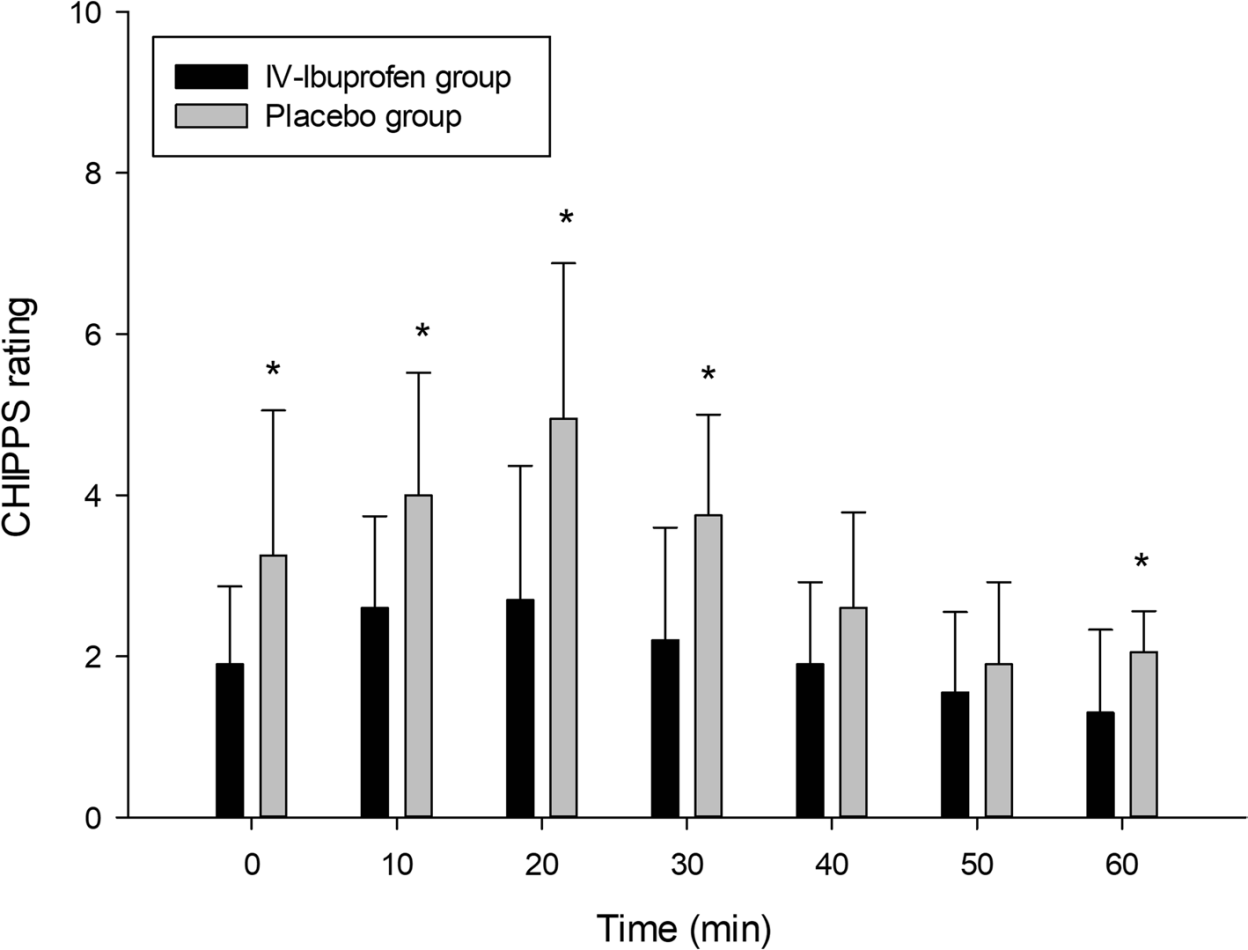
CHIPPS rating of two groups at different observational time points. CHIPPS, Children and Infants Postoperative Pain Scale; PACU = arrive at the recovery room immediately, further CHIPPS assessments at 10-min intervals; data are expressed as Mean ± SD; *n* = 20 in each group; *Indicates Mean significantly different between two groups

CHIPPS system was used to assess postoperative pain with or without fentanyl administration (Table 3). CHIPPS rating were significant at 0 min, 10 min, 20 min, 30 min and 60 min observational time points between two groups (*p*<0.001; Fig. 2). Titrated fentanyl treatment achieved satisfactory analgesia (CHIPPS < 4) after 30 to 40 min arrival at PACU in all patients.

**Table 3:** CHIPPS rating at different observation time points in PACU (mean ± SD)

Time (min)	0	10	20	30	40	50	60
IV-Ibuprofen group	1.90 ± 0.97	2.60 ± 1.14	2.70 ± 1.66	2.20 ± 1.40	1.90 ± 1.02	1.55 ± 1.00	1.30 ± 1.03
Placebo group	3.25 ± 1.80	4.00 ± 1.52	4.95 ± 1.93	3.75 ± 1.25	2.60 ± 1.19	1.90 ± 1.02	2.05 ± 0.51
*p*-value	0.005	0.002	<0.001	<0.001	0.089	0.280	0.006

*CHIPPS* Children and Infants Postoperative Pain Scale, *PACU* Postanesthesia care unit; *n* = 20 in each group

A total of 3 patients were reported agitation and they were all from Placebo group. In these patients, the symptom was gradually improved after treatment with fentanyl at preset rescue doses. The fentanyl administration in PACU was prudently titrated injection, while there was still one patient from Placebo group suffered from temporary oxygen desaturation ≤93% after three doses rescue application, the hypoxia was relieved after persistent oxygen therapy without prolongation of observation time. Bradycardia and respiratory depression ≤10 breaths/min were not occurred in the PACU. Intraoperative blood loss were 15.9 ± 8.9 ml (IV-Ibuprofen group) and 19.7 ± 9.2 ml (Placebo group) respectively. There was no difference in surgical blood loss (*p* = 0.194). Besides, there was no incidence of vomiting, nausea, and any adverse events within the first 24 h after surgery. No patient received a surgical re-exploration during hospitalization.

## Discussion

The major finding of this study was that for patients undergoing palatoplasty, received ibuprofen injection in a single dose of 10 mg/kg at induction of anesthesia contributed to significantly fewer doses and amount of postoperative fentanyl compared with placebo in early postoperative period. Besides, repeated administration of fentanyl should be carefully for this group although intravenous titrated fentanyl for analgesia produced reliable pain without discernible side effect at most of the time.

Acute postoperative pain is extremely common after surgical correction for velopharyngeal. Postoperative pain for palatoplasty mainly distribute in innervation area of the branches of maxillary division of trigeminal nerve [19]. The optimal analgesia strategy for infants continues to be a challenge. Reasonable analgesia results in less crying after operation that in turns decreases risk of wound dehiscence and postoperative bleeding. Patients could benefit from opioids treatment but related respiratory complication raises justifiable concerns, especially this group suffers from pharyngeal space reduction due to postoperative traumatic airway edema. Postoperative complete wellbeing of patients is also not just a pain free state regardless of potential risks. Thus, clinicians try to explore multimodal approach which seems to decrease opioids consumption with pain relief improvement [20–22]. Regional block anesthesia is becoming increasingly popular and the effect is positive, but it is difficult to identify intraneural placement of needle precisely in pediatric patients and to recognize block failure under general anesthesia [23, 24]. Besides, preincisional infiltration can also lead to esthetic repair difficult [21]. Meanwhile, NSAIDs are relatively convenient to be used as combination drugs for their potential opioid sparing effect.

Our study indicated the opioid sparing effect of ibuprofen injection. This finding remained similar with previous study in pediatric patients received tonsillectomy [14]. However, it should be noted that the analgesia effect of ibuprofen injection was seemed not always adequate and 8 (40.00%) patients from IV-Ibuprofen group received rescue analgesia therapy in PACU. The first rescue analgesia time between two groups was statistically insignificant. Thus, it might be suggested that the combination use of ibuprofen with opioids was a potential choice for severe postoperative pain therapy of intraoral surgery. Ibuprofen injection was a suitable adjuvant for pain treatment in our study.

CHIPPS system is effective in distinguishing true pain from other forms of perioperative discomfort such as anxiety and fear in infants. The assessment is easy to operate that can be completed within 15 s. This study used CHIPPS to demonstrate the effect of multimodal analgesia at different time point in PACU. In our study, CHIPPS rating between two groups were significant at arriving in the recovery room 0 min, 10 min, 20 min, 30 min and 60 min later respectively. The result suggested that the analgesia effect of multimodal approach was significant in the early postoperative period. Although all participants were treated with satisfaction (a pain score<4 of 10), the CHIPPS scores of participants from IV-ibuprofen group were significantly lower considering the interaction between group and observation time. Thus, combination of analgesia drugs therapy strategy was recommended for this group.

Compared to acetaminophen, ibuprofen is resulted to be more effective in practice according to the summary from available study [25]. Besides, it also has potential to limit the inflammatory cascade from surgical trauma and reduce the development of postoperative pain after this procedure compared with acetaminophen. Our research also suggested positive effect on postoperative analgesia. Furthermore, injectable formulation provided easy administration for patients suffered from postoperative dysphagia.

Clinicians are hesitant to use NSAIDs in procedure concerning the potential for hemorrhage. The result suggested insignificantly difference in surgical blood loss between groups. But this result was conservative because of hemocoagulase was routinely used intraoperative in study. One patient from Placebo group suffered from hypoxia after repeated use of rescue fentanyl, the symptom was relieved after persistent oxygen therapy, the medical history showed that the infant was suffered from recurring respiratory infections, and this was common for patients with cleft palate in clinic. Thus, repeated administration of fentanyl should be carefully for this group. No other adverse events were reported within the first 24 h after surgery.

Our study had several limitations. Firstly, fentanyl was allowed to use to facilitate intubation, which might influence the detective ability of opioid sparing effect for ibuprofen injection. This anesthesia protocol was moderatamente compared with similar studies previously [9, 11]. The second limitation was that ibuprofen administration was a single dose at induction to detect analgesia effect in early postoperative period. More trials might be undertaken to study the effect of drug accumulative administration after palate surgery. Moreover, we only reported limited opioid related respiratory complication in research, but it should be emphasized that some side effects such as pruritus was difficult to perceive in infants.

## Conclusions

The research indicated that preemptive administration ibuprofen injection in a single dose 10 mg/kg at induction for isolated cleft palate repair in infants had a significant opioid sparing effect in the early postoperative period without obvious adverse effects. Ibuprofen injection was an efficacious adjunct to fentanyl for treatment of postoperative acute pain, which could be considered as the first line medication for palatoplasty pain management regimen.

## Data Availability

The datasets used and/or analyzed during the current study are available from the corresponding author on reasonable request.

## Abbreviations

CHIPPS: Children and infants postoperative pain scale
CL: Cleft palate
NSAIDs: Nonsteroidal anti-inflammatory drugs
FDA: Food and Drug Administration
ASA: American Society of anesthesiologists
PACU: Postanesthesia care unit
SD: Standard deviations
IQR: Interquartile
CONSORT: Consolidated Standards of Reporting Trials
